# Childhood-onset dense deposit disease: a rare cause of proteinuria

**DOI:** 10.1007/s11845-013-1041-8

**Published:** 2013-12-14

**Authors:** K. Taranta-Janusz, A. Wasilewska, B. Szynaka

**Affiliations:** 1 Department of Pediatrics and Nephrology, Medical University of Białystok, Waszyngtona 17, 15-274 Białystok, Poland; 2 Cathedral of Biostructure Department of Pathomorphology, Medical University of Białystok, Białystok, Poland

**Keywords:** Dense deposit disease, Membranoproliferative glomerulonephritis, Child

## Abstract

**Introduction:**

Dense deposit disease (DDD) is a rare renal disease related to the dysregulation of the alternative pathway of the complement cascade, caused by several factors including the presence of an autoantibody to C3 nephritic factor, mutations in factor H and autoantibodies to this protein. DDD is characterized by C3 accumulation with absent or scanty immunoglobulin deposition.

**Case Presentation:**

Herein we report the case of a child with benign course of DDD, who presented with moderate proteinuria and lack of clinical symptoms without immunosuppressive treatment. Laboratory testing revealed moderate proteinuria, normal serum creatinine, total protein, and albumin levels, but significantly decreased serum C3 level. The results of renal biopsy were consistent with DDD. Genetic analysis revealed that the patient carried one copy of the H402 risk allele of factor H. The level of proteinuria did not change during the follow-up period and no nephrotic syndrome signs occurred. Renal function was stable.

**Conclusion:**

In conclusion, a program of urine screening for asymptomatic proteinuria and hematuria to detect children with kidney disease before they experience loss of kidney functions should be considered. Children diagnosed with DDD should have the opportunity to get treatment early on and to be followed very closely.

## Introduction

Dense deposit disease (DDD), also known as membranoproliferative glomerulonephritis type II (MPGN), is a rare disease. It primarily affects children and young adults. According to the new classification of MPGN proposed by Sethi et al. [1], DDD is a negative immunoglobulin and positive C3 glomerulopathy. It is mostly characterized by MPGN pattern of injury, C3 deposits on immunofluorescence (IF) microscopy and characteristic sausage-shaped, wavy deposits by electron microscopy inside the glomerular basement membrane (GBM) and mesangium [2, 3]. The clinical picture of the disease presents as acute nephritis, proteinuria or nephrotic syndrome. The long-term prognosis to retain native kidney function is usually poor. The pathogenesis of DDD is still obscure, however, it was shown that MPGN resulting from monoclonal gammopathy [4] and dysfunction of the alternative pathway (AP) of complement as the most frequently found factors. Recent discoveries strongly suggest that factor H (FH) plays a significant role in the pathogenesis of DDD [5, 6].

We report the case of a child with benign course of DDD, who presented with moderate proteinuria and lack of clinical symptoms without immunosuppressive treatment.

## Case report

Our female patient is the third child of non-consanguineous parents. The pregnancy and delivery had been uneventful. The child went on to have normal developmental milestones. Her past and family histories were not remarkable for renal diseases.

A previous healthy 8-year-old girl was referred to Department of Pediatrics and Nephrology, Medical University of Bialystok, Poland for proteinuria detected in a screening program at her elementary school. On admission, the girl was in good general condition (body weight 33.6 kg [75–90 pc], height 133 cm [75 pc], blood pressure 85/60 mmHg). Physical examination revealed no edema. She was normotensive. Admission laboratory investigations showed leukocyte count 6.7 × 10^3^/mm^3^, hemoglobin level 12.7 g/dL, hematocrit 37.9 %, platelet count 257 × 10^3^/mm^3^, serum creatinine 0.47 mg/dL, albumin 4.56 g/dL, cholesterol 260 mg/dL, C3 complement 63.1 mg/dL; C4 20.1 mg/dL, and ASO-tire 112 IU. Antinuclear antibody (ANA), and anti-neutrophil cytoplasmic antibody (ANCA) were negative. Urine protein excretion was 186–680 mg/24 h. The sediment contained 3–5 red blood cells per high power field (HPF). Serum immunofixation electrophoresis study for free light chains and urine electrophoresis for monoclonal gammopathy was negative. Glomerular filtration rate (GFR) measured by Schwartz formula was within the normal range (113 mL/min/1.73 m^2^). Ultrasonography of kidneys showed normal-sized kidney with normal corticomedullary distinction.

**Fig. 1:**
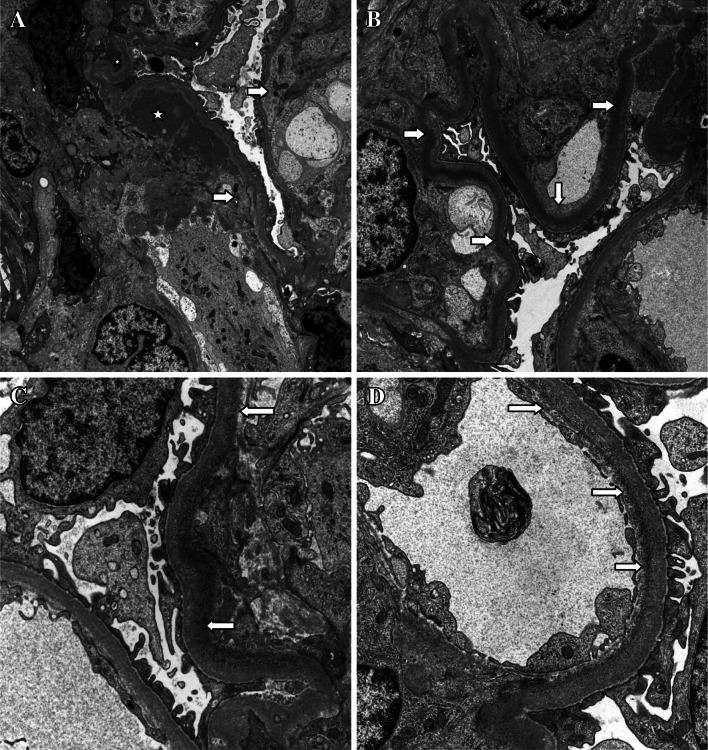
**a** Glomerular capillary walls with mesangial interposition (*rightwards double arrow*) and dense deposits at mesangial and subendothelial location (*asterisk*). Mag. ×7.000. **b** Segments of capillary wall with electron-dense material along the glomerular basement membrane which appears as a homogenous band. ME Mag. ×7.000. **c** The electron-dense deposits of the immune complexes with fine granules along one of the glomerular basement membrane under endothelium (*rightwards double arrow*). ME Mag. ×12.000. **d** The deposits of fine granular material along the glomerular basement membrane under endothelium (*rightwards double arrow*). ME Mag. ×12.000

The patient underwent ultrasound-guided renal biopsy. In the material examined under a light microscope, there were 16 glomeruli with a well-preserved structure. Only in six of them a slight segmental mesangial hypercellularity and an increased matrix mesangium were observed. In these glomeruli some capillaries showed double-contoured basement membrane after Jones’ silver stain and PAS stain and a thickening of the walls. No interstitial changes were found. The material for the IF examination contained 13 glomeruli. Moderate or scarce granular deposits of C3 (+2), IgG (+2), IgM (+1) (intensity on a scale of 0–4) located along the capillary walls of the glomeruli were found. Staining with immunoglobulin IgA was negative. Light-chain restriction was not documented in the IF microscopy examination. Electron microscopy was performed on six of the glomeruli. Ultrastructural examination revealed scattered amorphous, electron-dense deposits in the GBM under endothelium, in paramesangial and mesangial region, and occasionally with mesangial interposition (Fig. 1a). Fine granular osmiophilic, electron-dense material along the GBM which sometimes formed a homogenous elongated band under endothelium was also observed (Fig. 1b–d). These deposits appeared mainly in the peripherally placed capillaries and occasionally in mesangium matrix.

**Fig. 2:**
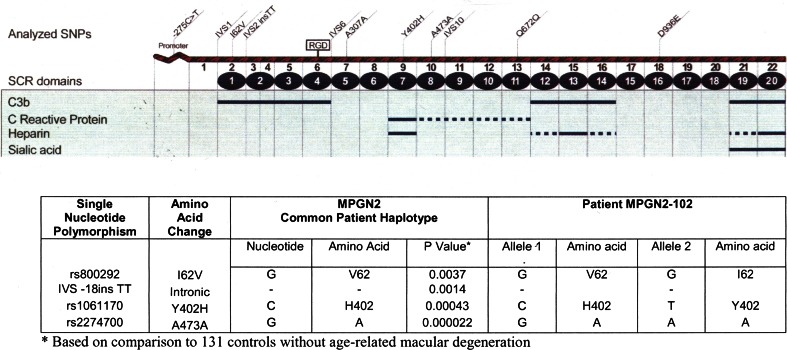
Factor H mutation screening of our patient

Genetic analysis was performed in the laboratory of the University of Iowa in the USA. No disease-causing mutations in the coding sequence or splice sites of factor I (FI) and membrane cofactor protein (MCP) were found. However, genetic testing for FH mutation established that the patient carried one copy of the H402 risk allele of FH (Fig. 2). FH-related proteins (CFHR3–CFHR1) testing revealed normal allele. No FI, MCP and FH gene mutations were found in the parents and siblings of the affected child.

During a follow-up period of 3 years, patient’s laboratory tests showed urine protein excretion 186–680 mg/24 h, slight erythrocyturia, decreased serum C3 (62–83.1 mg/dL), and normal kidney function. The patient was started on an angiotensin-converting enzyme (ACE) inhibitor (5 mg/day). Despite moderate proteinuria, she stayed clinically asymptomatic. After 3 years she was lost to follow up for about 1 year. During that time she discontinued medicines but continued to feel well. Laboratory tests revealed normal kidney function and moderate proteinuria.

## Discussion

This is a rare case of a child with DDD and specific polymorphism of the FH gene, who was diagnosed by a screening program. The girl was in a good general condition at admission and presented with moderate proteinuria and lack of clinical symptoms without immunosuppressive treatment. To date few cases of DDD in children with spontaneous remission have been reported and most of them were treated with steroids [7]. In most of the cases, the disease leads to chronic kidney disease. Lu and colleagues [8] who surveyed 98 DDD patients and their families noted that half of the estimated patients progressed to end-stage renal disease (ESRD) with greater risk for renal failure in females.

Typical features of DDD include the complement profile and the presence of C3 nephritic factor. The profile usually includes low C3 levels with normal levels of other components. Similar profile of complement was found in our patient. The relationship among C3 and C3NeF levels and prognosis is still unclear. Nasr et al. [9] have not found any correlation, whereas other authors confirmed that low complement level is a predictive factor of poor prognosis [2].

Understanding of the genetics of DDD is far from complete. The disease is classified as an autoimmune disorder primarily because of the frequent finding of circulating IgG antibodies known as C3NeF that is directed against the C3 convertase, thereby, hindering its degradation. The result of delayed degradation is unchecked activation of the AP, with the consumption of serum C3. Of the genes associated with DDD, the most robust data are available for FH. FH is the primary fluid-phase regulator of the AP of complement. The importance of FH is reflected by the fact that its total absence in mice and pigs leads to unchecked C3 convertase activity, excessive activation of the alternative complement pathway and eventual development of DDD. Similar outcome is seen in humans. Licht et al. [10] described deletion, ΔK224 in two siblings with DDD that resulted in the omission of one amino acid in FH protein and as a consequence, impaired the ability of FH to bind to C3b and inactivate the C3 convertase of the AP. Similarly, an association study showed that H402 allele of FH was found in 85 % of persons with DDD and much less frequently in general population. The H402 allele variant of FH results from a single nucleotide polymorphism which changes the codon encoding the amino acid tyrosine to one encoding histidine. Studies of the H402 allele show that binding of FH to the GBM may be important in protecting it from complement-mediated damage by the AP and that binding of the H402 allele of FH to the GBM is compromised. The study of Leroy et al. [11] performed on eight patients diagnosed with idiopathic MPGN revealed a low C3 level with normal values of FH, FI and surface expression of MCP with genetically confirmed heterozygous mutation of FH, similar to the results of our patient. Boon and co-workers [12] described in their study that individuals who carry FH mutation on one allele in combination with the presence of the H402 on the other allele developed age-related macular degeneration (AMD). Servais et al. [13] found that genetic background of the patients may also influence the disease manifestation since common genetic variants including single nucleotide polymorphisms in the FH gene are associated with DDD.

In contraposition of MPGN I, there was no constant glomerular finding by light microscopy with a variation of microscopic pattern. Only 6 of 16 of the glomeruli presented focal segmental mesangial proliferation and occasional thickening of the GBM with the double-contoured basement membrane. So, picture in light microscope supports DDD. However, IF study was rather typical for MPGN I with deposits of C3 complements (+2), IgG (+2) and IgM (+1), instead of typical for DDD striking C3 positivity along the capillary loops.

The hallmark of ultrastructural examination that supports diagnosis of immune complexes disease (MPGN) is the presence of mesangial, paramesangial and subendothelial electron-dense deposits as well as fine granular material along the GBM under endothelium. These linear deposits might suggest light-chain deposition disease (LCDD), but do not correspond with IF. These deposits are also seen in early stages of DDD in children. Deposits in DDD usually present more as a homogenous than granular, and showed ribbon-like pattern within the GBM. Localization of deposits in peripheral capillary loops with results of IF study and negative light-chain restriction predispose to DDD diagnosis.

Sethi et al. [1] found that 41 % of patients with DDD had serum and/or urine electrophoresis studies positive for monoclonal gammopathy. The authors suggested that DDD may often be the first sign of the underlying lymphoplasmacytic disorder. It was found that DDD might be the complication of monoclonal gammopathy of undetermined significance. In this base the monoclonal Ig acts as an autoantibody to FH or other complement regulating proteins that on a permission genetic background (the H402 allele of FH) leads to dysregulation of the AP with subsequent MPGN. We looked for monoclonal gammopathy in our patient, however, the electrophoresis was negative and no clinical signs were observed. We wish we used serum immunofixation electrophoresis method which is more sensitive.

The prognosis of patients with DDD is generally quite poor, especially in patients with nephritic syndrome, who do not respond to treatment. Although there are some reports of spontaneous remissions of DDD, this is still uncommon, as most of the patients progress to ESRD [14]. Specific mutations and variants in the FH gene are associated with broad range of phenotypes, from early onset renal diseases with high mortality rates to disorders limited to the eye [15]. Differences in clinical phenotypes between our patient and others might depend on variants in the FH gene. The H402 allele of FH is very frequent in the population. There is a higher frequency in AMD and also in C3 glomerulopathy, but this variant alone is not responsible for the complement overactivation in DDD. Severity of clinical features may depend partly on specific FH sites affected by mutation. We suppose that another reason for a stable clinical course might be the absence of detectable abnormalities of other factors influencing systemic alternative complement pathway activation, i.e., C3Nef, FI. However, direct influence of this gene abnormality upon our patient’s renal condition remains unclear. Further studies are necessary to find out whether a particular clinical phenotype or histopathology pattern predicts response to therapy who should be treated and who will have a relatively benign disease course.

In conclusion, a program of urine screening for asymptomatic proteinuria and hematuria to detect children with kidney disease before they experience loss of kidney functions should be considered. Children diagnosed with DDD should have the opportunity to get treatment early on and be followed very closely.
